# Retinal arterial tortuosity in Ehlers–Danlos syndromes

**DOI:** 10.1038/s41433-022-02278-x

**Published:** 2022-10-14

**Authors:** Hashem H. Ghoraba, Darius M. Moshfeghi

**Affiliations:** Byers Eye Institute, Stanford University School of Medicine, Palo Alto, CA USA

**Keywords:** Retinal diseases, Hereditary eye disease

## Abstract

**Purpose:**

To report a novel finding of retinal arterial tortuosity (RAT) associated with Ehlers–Danlos syndromes (EDS).

**Methods:**

We queried the STAnford Research Repository (STARR) database to identify patients diagnosed with EDS. We included patients with a confirmed diagnosis of any subtype of EDS who had any form of readable retinal imaging including colour fundus photos, autofluorescence, red-free photos, red-free optical coherence tomography photos and fluorescein angiography. Patients who had no retinal imaging and those with no confirmed EDS diagnosis were excluded. Retinal images were reviewed for RAT and were graded into no, possible and definite RAT. Eyes with definite RAT were further graded into mild, moderate and severe. Eyes with definite RAT were again subclassified according to the type of involved vessels into first-order arteriolar, macular and arteriovenous.

**Results:**

A total of 307 patients were identified using the STARR tool and 142 patients were included. Mean age was 40.9 ± 18.1 years and 87% were female. Underlying EDS subtypes were hypermobile EDS (69.7%), classical EDS (2.8%), vascular EDS (2.1%), myopathic EDS (0.7%) and not specified (24.6%). We graded 37.3% of patients with definite RAT, 10.6% with possible RAT and 52.1% with no RAT. In patients with definite RAT, we graded 39.2% of eyes with mild RAT, 40.2% with moderate RAT and 20.6% with severe RAT. In all, 84.9% showed involvement of first-order retinal arterioles, 35.8% showed involvement of macular arterioles and 1.9% showed arteriovenous involvement.

**Conclusions:**

Variable degrees of RAT are associated with EDS.

## Introduction

Ehlers–Danlos syndromes (EDS) are a group of heterogeneous connective tissue disorders that occur secondary to defective collagen synthesis. They are classified into 13 clinical subtypes with common features of joint hypermobility, skin hyperextensibility and tissue fragility [1].

The prevalence rate of EDS is not clear. Some studies estimated an overall prevalence of 1:5000 for all subtypes of EDS [1]. Other studies have reported significantly higher rates up to 10:5000 [2]. No sex predilection has been observed for all subtypes, except for the hypermobile subtype of EDS, which is reported to be more prevalent in females [1–3]. The most common subtypes of EDS are the hypermobile (hEDS), classical (cEDS) and vascular (vEDS) [3].

The classification of EDS is based on major and minor clinical criteria, with significant overlap between different subtypes. cEDS is mainly characterized by skin hyperextensibility and generalized joint hypermobility, in addition to other minor criteria such as easy bruising and skin fragility. hEDS is mainly characterized by generalized joint hypermobility, generalized connective tissue disorder, and evidence of musculoskeletal complications, but with the absence of unusual skin fragility. vEDS is characterized by positive family history and/or genetic testing and spontaneous arterial or organ rupture at a young age in addition to minor criteria such as easy bruising and translucent skin [4]. All 13 clinical subtypes have been associated with identified genetic abnormalities except for hEDS, whose genetic basis has not been identified [3, 4]. Except for hEDS, the 2017 International Classification of the Ehlers–Danlos Syndromes [4] recommended performing genetic testing of EDS to confirm and distinguish different subtypes. hEDS diagnosis is still based solely on clinical presentation. EDS are inherited based on autosomal dominant, recessive patterns, or both, according to the subtype [4].

The pathophysiology of EDS differs according to the subtypes. This includes defects in fibrillar pro-collagen types I, III and V; defects in collagen cross-linking and folding; defects in glycosaminoglycans biosynthesis; defects in extracellular matrix (ECM) bridging molecules; and other rare defects involving intracellular molecules and complementary pathways [3].

Abnormal blood vessels are a key feature of some EDS subtypes, especially vEDS. Spontaneous arterial rupture, including the aorta, is a potential cause of morbidity and mortality. Aneurysmal formation and arterial dissection can occur. Early onset varicose veins are also associated with vEDS [3, 4]. Systemic arterial tortuosity has been reported in association with vEDS [5, 6]. Vertebral arterial tortuosity has been suggested as a biomarker for vascular events in the young population with vEDS [6].

Ocular manifestations in EDS are variable and include blue sclera, pathologic myopia, steep and thin corneas, keratoconus, keratoglobus, retinal detachment and dry eye syndrome [4, 7, 8]. We noted that our index patient diagnosed with EDS had retinal vascular tortuosity affecting the arteries. This study aims to describe our findings.

## Methods

### The setting of the study and subjects

This was a retrospective, observational study in which we queried the Stanford Research Repository (STARR) tool to identify patients with the diagnosis of EDS, using ICD code Q79.6, who were seen at Byers Eye Institute at Stanford. We performed a chart review and included patients who had a confirmed diagnosis of any subtype of EDS and had any form of retinal imaging in the Zeiss Forum Viewer at any timepoint including colour fundus photos (FP), autofluorescence (AF), red-free photos (RF-FP), red-free optical coherence tomography (OCT) photos and fluorescein angiography (FA) as available. Patients who had no form of retinal imaging and those with no confirmed EDS diagnosis were excluded.

The study was conducted in compliance with the Declaration of Helsinki, the United States Code of Federal Regulations Title 21, and the Harmonized Tripartite Guidelines for Good Clinical Practice (1996). Stanford University institutional review board approved the study under protocol 36544, and an informed consent waiver was obtained as the charts of enrolled patients were retrospectively reviewed.

### Data collection and outcomes

We collected demographic data, subtype of EDS and genetic testing results. Retinal images including FP, RF-FP, red-free OCT, AF and FA were utilized for grading of retinal arterial tortuosity (RAT). Two graders (DMM and HHG) reviewed all available retinal images for RAT and graded them into no, possible and definite RAT. Possible RAT was determined by a disagreement between the two graders regarding the presence of RAT. Eyes with definite RAT were further graded into mild, moderate and severe. We defined mild and severe RAT as vascular tortuosity definitely similar to pre-plus and plus disease in retinopathy of prematurity, respectively. We defined moderate RAT as an intermediate degree of tortuosity that would not fit to be mild or severe. Images with mild, moderate and severe tortuosity were then subclassified according to the type of involved vessels into first-order arteriolar, macular and arteriovenous. The subclassifications were not mutually exclusive. Further disagreements between the two graders regarding the degree and subclassification of RAT were resolved by discussion.

### Statistical analysis

Descriptive statistics were calculated for the variables of interest. Continuous variables were expressed in mean and standard deviation. Pearson’s *χ*^2^ test and Fisher’s exact tests were used to compare frequencies between different groups. One-sided Welch’s *t*-test was used to compare means between different groups. Cohen’s Kappa was used to assess intergrader reliability.

Data analysis was performed using RStudio® software (Version 1.3.1093).

## Results

### Demographic and baseline criteria

A total of 307 patients were identified using the STARR tool and 142 patients were included. In all, 148 patients were excluded due to the absence of readable retinal images and additional 17 patients were excluded due to the lack of diagnostic support for EDS.

Mean age was 40.9 ± 18.1 years and 87% were female. Underlying EDS subtypes were hEDS (69.7%, *n* = 99), cEDS (2.8%, *n* = 4), vEDS (2.1%, *n* = 3), myopathic EDS (0.7%, *n* = 1) and not specified (24.6%, *n* = 35).

Genetic testing was done in 27 patients and was positive for EDS-specific mutations in 6 patients, including 3 patients with vEDS (COL3A1 mutation), 2 patients with cEDS (one patient with COL5A1 mutation, and the other was not specified) and 1 patient with myopathic EDS (COL12A1) mutation. The remaining 21 patients who performed genetic testing were negative or inconclusive and were eventually diagnosed with hEDS.

### Grading of eyes with RAT

**Fig. 1:**
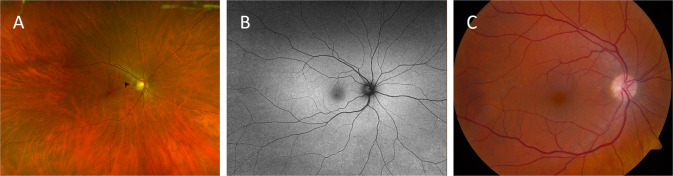
Representative retinal images of different eyes graded with possible retinal arterial tortuosity. **A**, **C** Colour fundus photos. Note the retinal yellowish-white dots (black arrowhead in **A**). **B** Retinal red-free image.

We graded 53 patients (37.3%) with definite RAT, 15 (10.6%) with possible RAT (see Fig. 1 for representative images) and 74 (52.1%) with no RAT. There was a substantial agreement between the 2 graders regarding the presence or absence of RAT (Kappa = 0.78). There were no statistically significant differences in age, sex or EDS subtype between patients with possible or definite RAT and those without RAT (Supplementary Table 1). Hypertension was present in 9.4% and 10.8% of patients with definite RAT and with no RAT, respectively, and the difference was not statistically significant (*P* = 0.9). Diabetes mellitus was present in 3.8% and 5.4% of patients with definite RAT and with no RAT, respectively, and the difference was not statistically significant (*P* = 0.95). None of these patients had any evidence of hypertensive or diabetic retinopathy.

**Fig. 2:**
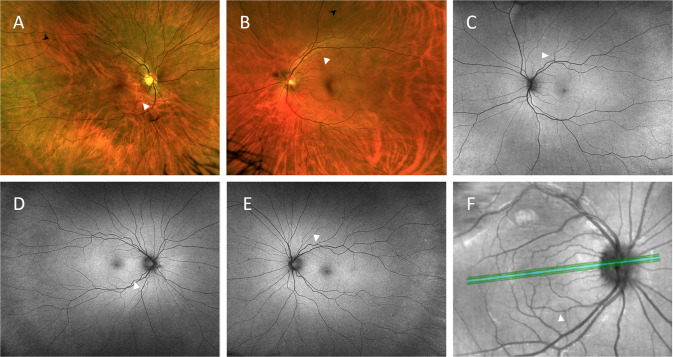
Representative retinal images of different eyes graded with mild retinal arterial tortuosity (white arrowheads). **A**, **B** Colour fundus photos. Note the retinal yellowish-white dots (black arrowheads). **C**–**E** Retinal red-free images. **F** Optical coherence tomography red-free image.

**Fig. 3:**
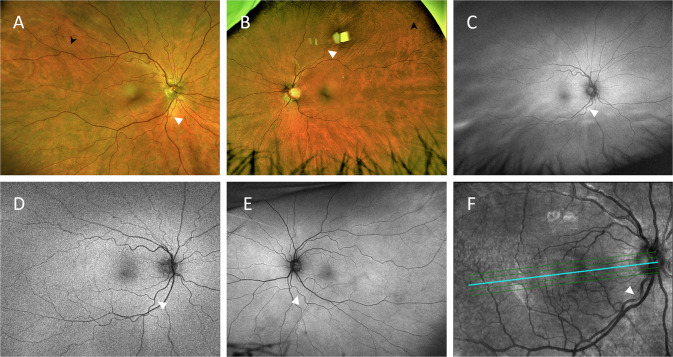
Representative retinal images of different eyes graded with moderate retinal arterial tortuosity (white arrowheads). **A**, **B** Colour fundus photos. Note the retinal yellowish-white dots (black arrowheads). **C**–**E** Retinal red-free images. **F** Optical coherence tomography red-free image.

**Fig. 4:**
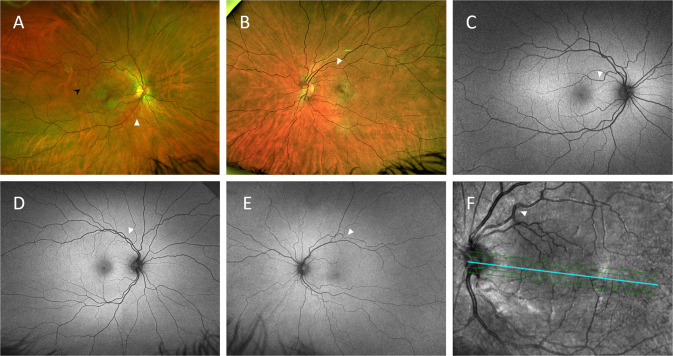
Representative retinal images of different eyes graded with severe retinal arterial tortuosity (white arrowheads). **A**, **B** Colour fundus photos. Note the retinal yellowish-white dots (black arrowhead in **A**). **C**–**E** Retinal red-free images. **F** Optical coherence tomography red-free image.

In patients with definite RAT (*n* = 53), 102/106 eyes were graded; 3 eyes did not show RAT and 1 eye did not have gradable images. Breakdown of eyes with definite RAT was as follows: 40 eyes (39.2%) graded with mild RAT (see Fig. 2 for representative images), 41 eyes (40.2%) graded with moderate RAT (see Fig. 3 for representative images) and 21 eyes (20.6%) graded with severe RAT (see Fig. 4 for representative images). There was a substantial agreement between the 2 graders regarding the degree of RAT (Kappa = 0.61). Severe RAT was not associated with the diagnosis of systemic hypertension or diabetes mellitus.

### Subclassification of eyes with RAT

**Fig. 5:**
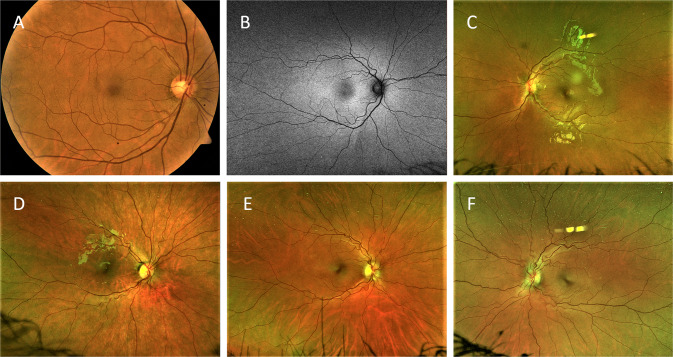
Representative retinal images of different eyes graded with different subclassifications of retinal arterial tortuosity (RAT) according to the involved vessels. **A** Colour fundus photo showing macular moderate RAT. **B**, **C** Red-free and colour fundus photos showing large arteriolar and macular severe RAT. **D** Colour fundus photo showing large arteriolar severe RAT. **E**, **F** Colour fundus photos showing large arteriovenous tortuosity and macular severe RAT.

We also subclassified patients with definite RAT according to the type of involved vessels. A total of 84.9% showed involvement of the first-order arterioles, 35.8% showed involvement of macular arterioles and 1.9% showed arteriovenous involvement. Figure 5 shows representative retinal images subclassified according to the involved vessels.

### Other findings

Scattered random yellowish-white retinal dots were noted in 52.9% of patients (*n* = 36; 70 eyes) with possible and definite RAT (see Figs. 1–4). The nature and clinical significance of these yellowish-white dots are not clear.

## Discussion

In the index study, we noted that a significant portion of patients with EDS demonstrated RAT, which, to our best knowledge, has not been previously reported. Nearly 50% of our cohort was graded with different degrees of RAT.

The most common underlying EDS subtype in our cohort was hEDS which is diagnosed clinically and does not require genetic testing [4]. This rationalizes the relatively few EDS genetic tests performed in our cohort. We could not explore potential differences in RAT between different subtypes due to the few numbers of patients diagnosed with subtypes other than hEDS.

To the best of our knowledge, hEDS is not known to be associated with significant vascular abnormalities. However, it is associated with postural orthostatic tachycardia syndrome (POST) that is explained by different mechanisms [9, 10]. One proposed mechanism is increased vascular extensibility, secondary to abnormal vascular structure and connective tissue, leading to increased venous pooling during the upright position [10–12]. Wandele et al. reported a strong linear relationship between skin extensibility and POST in patients with hEDS. They also noted that, when compared with other factors, skin extensibility was the most predictive for POST [10]. This finding suggests that hEDS is associated with hyperextensible blood vessels secondary to abnormal vascular connective tissue and ECM. This can explain the findings in our cohort, in which retinal arterioles with abnormal vascular ECM become torturous.

Different diseases are associated with retinal vascular tortuosity. The most common causes are retinal vein occlusion, hypertensive retinopathy, diabetic retinopathy, carotid-cavernous fistula, and retinopathy of prematurity [13, 14]. The mechanism of retinal vascular tortuosity is not well understood and is probably different according to the underlying cause. One proposed mechanism is increased back pressure, as in cases of retinal vein occlusion and carotid-cavernous fistula. Vascular endothelial growth factor has been hypothesized to cause retinal vascular tortuosity in cases of diabetic retinopathy and retinopathy of prematurity [14, 15]. Hypertensive retinopathy has been shown in some studies to be associated with tortuous retinal arterioles that was suggested to be secondary to increased retinal blood flow [13]. In our cohort, the presence of hypertension or diabetes mellitus (10% and 3%, respectively) cannot explain the association between RAT and EDS.

Familial retinal arterial tortuosity (FRAT) is an autosomal dominant condition characterized by tortuosity of second and third-order retinal arterioles, especially in the macular region, and typically sparing larger first-order retinal arterioles [16, 17]. FRAT has been recently associated with mutations in *COL4A1* gene which encodes for type 4 collagen present in basement membranes of different tissues, including retinal blood vessels [17]. FRAT can be asymptomatic or be associated with retinal haemorrhage that ensues spontaneously or following minor trauma [18, 19]. FRAT usually presents without systemic association but some patients have presented with different systemic vascular abnormalities, including cerebral aneurysms [20] and hereditary angiopathy with nephropathy, aneurysm, and muscle cramps [21], both of which were associated with *COL4A1* mutations; and familial haematuria that was associated with basement membrane abnormalities [22]. Many of the 13 clinical subtypes of EDS are associated with different COL mutations, including *COL1A1*, *COL1A2*, *COL3A1*, *COL5A1* and *COL12A1*, which in turn lead to abnormal collagen and ECM [4]. Different subtypes of EDS, other than vEDS, are associated with vascular abnormalities [23].

In contrast to FRAT, most of our cohort (84.9%) demonstrated RAT in the first-order arterioles. Macular involvement was limited to 35.8%. None of our cohort presented with, or had a history of, spontaneous or post-traumatic retinal haemorrhage. These discrepancies between the observed phenotype in our cohort and FRAT suggest that their pathogenesis is different, although both probably occur secondary to vascular ECM abnormalities.

Another interesting finding in our cohort was the frequently observed retinal yellowish/white dots. It is unlikely that these dots are secondary to a vascular aetiology but could represent a degenerative process related to the underlying pathology of EDS.

The limitations of our study include the retrospective nature, which increases the possibilities of unknown confounders. However, a detailed chart review of all of our cohorts did not show systemic or ocular diseases that would explain our findings. The absence of a control group of the normal population should also be considered a limitation, although it is expected that a normal retina would not show any evidence of RAT. Another limitation is that the underlying EDS subtype was mostly hEDS, which is diagnosed based on clinical, rather than genetic, criteria and can overlap with other hypermobility disorders. The limited number of patients with other subtypes of EDS has not allowed us to explore variations in RAT and other possible vascular changes amongst different EDS subtypes. In addition, a significant portion of our cohort (about 25%) did not have a specified subtype of EDS.

## Conclusion

A significant portion of patients with EDS demonstrated various degrees of RAT that is distinct from FRAT. Larger multicenter studies are required to fully elucidate our observation and explore its clinical significance.

## Summary

### What was known before

- Ehlers–Danlos syndromes were associated with different ocular manifestations including the blue sclera, pathologic myopia, steep and thin corneas, keratoconus, keratoglobus and dry eye syndrome.

### What this study adds

- In this study, we demonstrate that Ehlers–Danlos syndromes are associated with variable degrees of retinal arterial tortuosity.

## Supplementary information

Supplementary Table 1

## Data Availability

The data that support the findings of this study are available on request from the corresponding author [DMM]. The data are not publicly available due to them containing information that could compromise research participant privacy/consent.
