# Supplementary material for: Retinal arterial tortuosity in Ehlers–Danlos syndromes
Source: Eye (Lond). 2022 Oct 14;37(9):1936–41. doi: 10.1038/s41433-022-02278-x (PMC10275944; doi:10.1038/s41433-022-02278-x)
Supplement: Supplementary file 1 — Supplementary Table 1 [file 41433_2022_2278_MOESM1_ESM.docx]

| Table 1: Baseline differences between eyes with definite or possible RAT and those without RAT | | | |
| --- | --- | --- | --- |
|  | **Possible or definite RAT**  **(n = 68)** | **No RAT**  **(n = 74)** | **P value** |
| Age (mean ± SD) years | 40.7 ± 18.1 | 39.7 ± 16.4 | 0.7 |
| Sex (% female) | 87 % | 88 % | 1.0 |
| EDS subtypes |  |  | 0.6 |
| Hypermobile | 67.6 % (n = 46) | 71.6 % (n = 53) |  |
| Classical | 1.5 % (n =1) | 4 % (n = 3) |  |
| Vascular | 2.9 % (n = 2) | 1.4 % (n = 1) |  |
| Myopathic | 0 | 1.4 % (n = 1) |  |
| Not specified | 27.9 % (n = 19) | 21.6 % (n = 16) |  |
| RAT: retinal arterial tortuosity; EDS: Ehlers-Danlos syndrome. | | | |
